# Anti-cancer potential of flavonoids: recent trends and future perspectives

**DOI:** 10.1007/s13205-013-0117-5

**Published:** 2013-02-12

**Authors:** Priya Batra, Anil K. Sharma

**Affiliations:** Department of Biotechnology, MMEC, Maharishi Markandeshwar University, Mullana, Ambala, Haryana 133207 India

**Keywords:** Flavonoids, Anti-cancer, Breast cancer, Therapeutic potential, Health benefits, Dietary flavonoids

## Abstract

Cancer is a major public health concern in both developed and developing countries. Several plant-derived anti-cancer agents including taxol, vinblastine, vincristine, the campothecin derivatives, topotecan, irinotecan and etoposide are in clinical use all over the world. Other promising anti-cancer agents include flavopiridol, roscovitine, combretastatin A-4, betulinic acid and silvestrol. From this list one can well imagine the predominance of polyphenols, flavonoids and their synthetic analogs in the treatment of ovarian, breast, cervical, pancreatic and prostate cancer. Flavonoids present in human diet comprise many polyphenolic secondary metabolites with broad-spectrum pharmacological activities including their potential role as anti-cancer agents. A positive correlation between flavonoids-rich diet (from vegetables and fruits) and lower risk of colon, prostate and breast cancers lead to a question that whether flavonoids mediate the protective effects as chemopreventive agents or can interact with different genes and proteins to play role in chemotherapy. The current review emphasizes onto the therapeutic potential of flavonoids and their synthetic analogs as anti-cancer agents by providing new insights into the factors, regulation and molecular mechanisms along with their significant protein interactions.

## Introduction

Cancer chemoprevention, by the use of natural, dietary or synthetic agents that can reverse, suppress or prevent carcinogenic progression, has become an appealing strategy to combat the dogma associated with increasing cases of cancers worldwide (Tsao and Edward 2004). Epidemiological studies point to the fact that long-term consumption of diet rich in foods and vegetables reduces the risk of chronic diseases especially cancer (Wallstrom et al. 2000; Parr and Bolwell 2000; Arts and Hollman 2005; Patel 2008; Xiao et al. 2011). Such diets can minimize—exposure to deleterious substances, activation of procarcinogens and can maximize the intake of certain beneficial nutrients like isothiocyanates, unsaturated fatty acids, polyphenolic terpenoids (PPT), selenium, terpenes, etc. (Franco et al. 2004; Johnson 2004).

### Historical perspective

In 1930, a new substance was isolated from oranges that can reduce the capillary permeability and is believed to be a member of a new class of vitamins hence designated as vitamin P, however, later on this substance was identified as a flavonoid (rutin). Flavonoids drew greater attention with the decreased incidence of cardiovascular diseases, in spite of a greater saturated fat intake in Mediterranean population, which was associated with red wine consumption (Renaud and de Lorgeril 1992). Flavonoids belong to a very vast group of plant secondary metabolites with variable phenolic structures and are found in fruits, vegetables, grains, bark, roots, stems, flowers, tea and wine (Nijeveldt et al. 2001). In plants, flavonoids are performing a variety of functions including pollination, seed dispersal, pollen tube growth, resorption of mineral nutrients, tolerance to abiotic stresses, protection against ultraviolet and allelopathic interactions, etc. (Gould and Lister 2005; Samanta et al. 2011; Hassan and Mathesius 2012). More than 8,000 different compounds of polyphenols have been known and that can be further subdivided into ten different general classes (Ververidis et al. 2007; Harborne and Williams 2000; Chahar et al. 2011). Flavonoids are part of this family and have more than 4,000 varieties (Harborne 1994). Isoflavonoids (phytoestrogens or non-steroidal estrogens) such as the soy isoflavones—genistein and daidzein, have also been known for their therapeutic significance particularly in the protection of human health (Wiseman et al. 2000; Stevens and Page 2004; Ørgaard and Jensen 2008; Xiao 2008; Ogbuewu et al. 2010; Wiseman et al. 2002). There are a variety of factors such as species, variety, climate, degree of ripeness and post harvest storage which influence the concentration of flavonoids in foods (Holland et al. 1995; Robards and Antolovich 1997; Pascual-Teresa et al. 2000; Modak et al. 2011). Flavonoids have a remarkable reducing ability and ability to interact with proteins (Haslam 1996; Havsteen 2002; Liu et al. 2010; McRae and Kennedy 2011). This review focus on biochemical studies carried out to analyze the possible health effects of flavonoids and to assess their potential in the prevention of degenerative diseases or their therapeutic value as potential drugs.

### Structural insight and classification

Polyphenolic terpenoids are the most extensively studied flavonoids which have a characteristic C6–C3–C6 structure. The chemical structure of flavonoids is based on a C_15_ skeleton with a CHROMANE ring bearing a second aromatic ring B in position 2, 3 or 4.
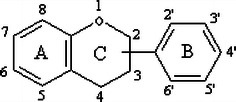


In a few cases, the six-membered heterocyclic ring C occurs in an isomeric open form or is replaced by a five-membered ring.
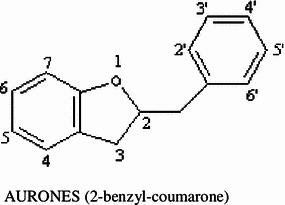


**Fig. 1:**
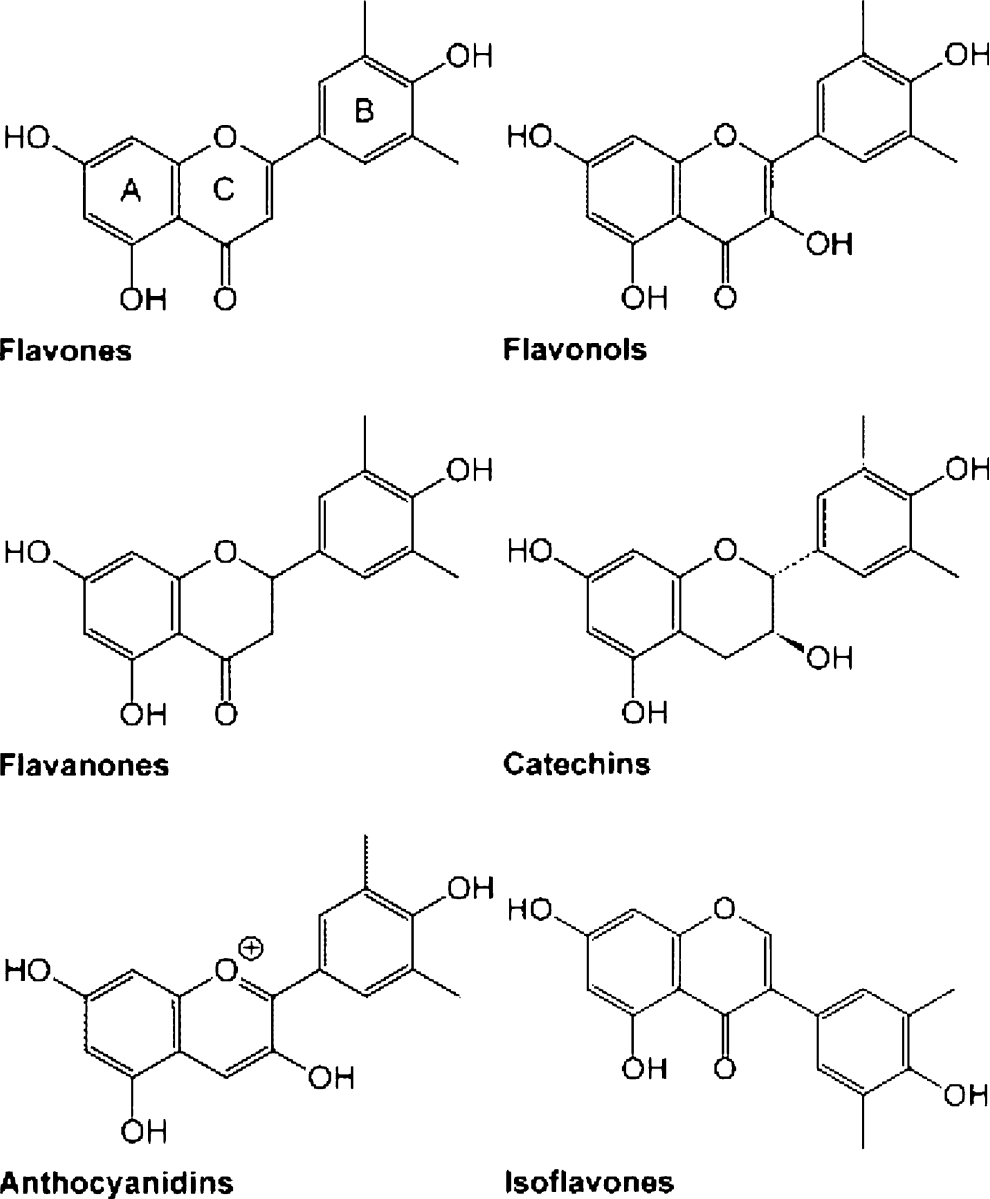
Subclasses of flavonoids

They have been further subdivided into flavones, flavonols, flavanones, flavanols, anthocyanins and isoflavones based on the nature of C3 element (Fig. 1). Different groups of flavonoids and their dietary sources are mentioned in Table 1. Flavonoids, especially flavanols, flavonols and anthocyanins are relatively abundant in human diet and possibly involved in prevention of cancers, cardiovascular diseases and neurodegeneration (Bazzano et al. 2002; Clifford 2004; Atmani et al. 2009; Fang et al. 2010; Xiao et al. 2011).

**Table 1:** Different groups of flavonoids and their dietary sources

Flavonoid group	Subgroup	Major sources	Anti-cancer properties
Flavanols	Flavan-3-ols: catechin, gallocatechin, catechin-3-gallate, epicaechin, epigallocatechin Flavan-4-ols Flavan-3,4-diols	Chocolate, green and black tea, beans, cherry, strawberries, cocoa, apple	Human oral, rectal and prostate cancer
Flavones	Apigenin, chrysin, luteolin	Parsley, celery, capsicum, pepper, broccoli	Lung cancer, leukemia, stomach, colon, thyroid, oral and laryngeal cancer, breast cancer
	Flavonol: kaempherol, myricetin, quercetin, rutin	Brussel sprouts, apples, onion, curly kale, leek, beans, cherries	
	Flavanones: eriodictyol, hesperitin, naringenin	Orange juice, grape fruit juice, lemon juice	
	Flavanonols: taxifolin	Milk thistle, red onion, acai palm, Siberian larch tree	
Anthocyanidins	Cyanidin, delphinidin, malvidin, petunidin, peonidin, pelargonidin	Aubergine, black berries, black currant, blue berries	Colorectal cancer
Isoflavonoids	Isoflavones: daidzein, genistein, glycitein Isoflavane: equol	Soy flour, soy beans, soy milk, miso, tempeh, beer Metabolized from daidzein by intestinal bacteria	Breast cancer, prostate cancer, colon, kidney and thyroid cancer

### Synthesis

**Fig. 2:**
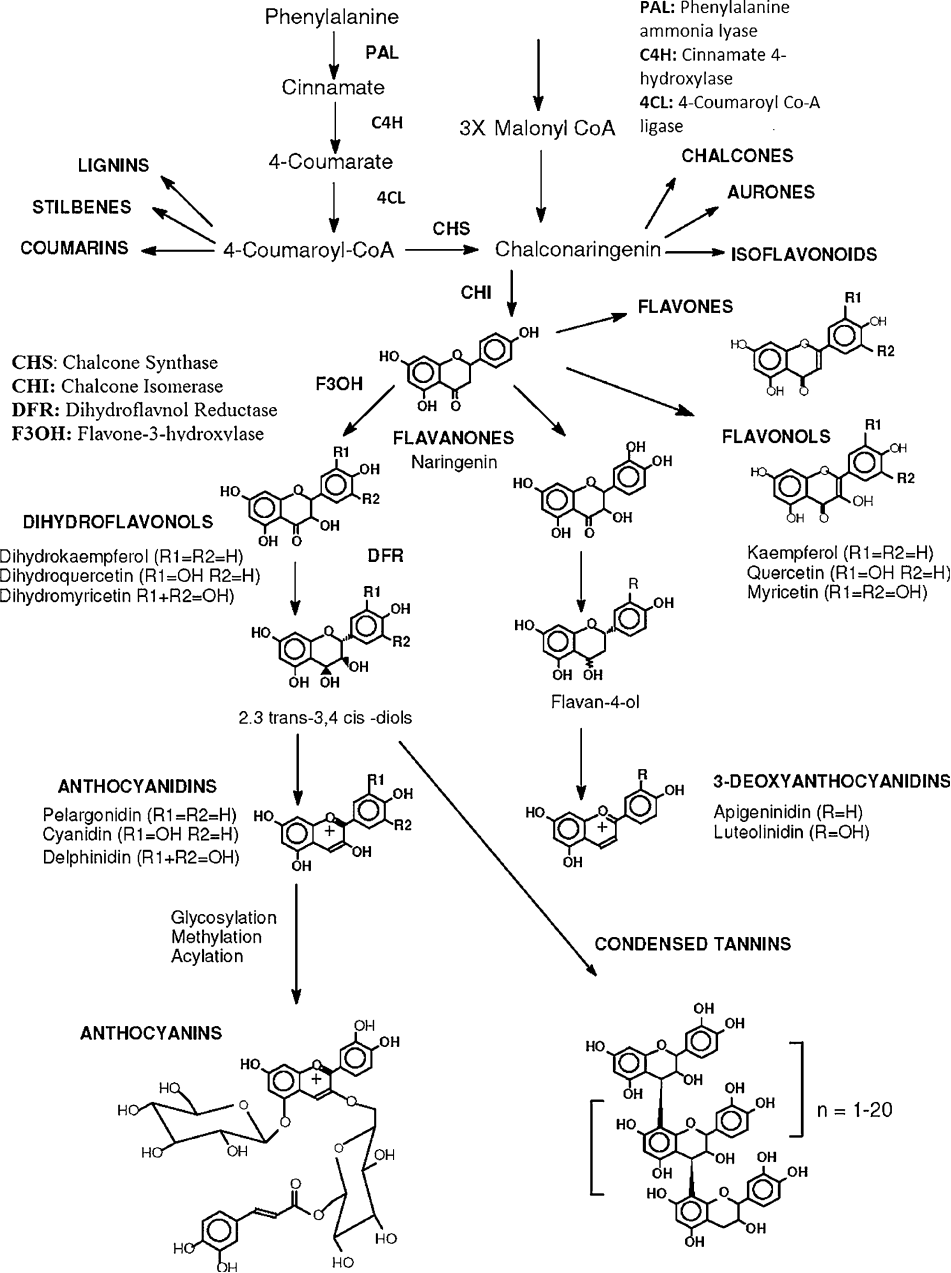
Pathway of biosynthesis of flavonoids

The flavonoids are formed in plants and participate in the light-dependent phase of photosynthesis during which they catalyze electron transport (Das 1994). They are synthesized from the aromatic amino acids—phenylalanine and tyrosine, together with acetate units (Heller and Forkmann 1993). Phenylalanine and tyrosine are converted to cinnamic acid and parahydroxycinnamic acid, respectively, by the action of phenylalanine and tyrosine ammonia lyases (Wagner and Farkas 1975). Cinnamic acid (or parahydroxycinnamic acid) condenses with acetate units to form the cinnamoyl structure of the flavonoids (Fries rearrangement). A variety of phenolic acids, such as caffeic acid, ferulic acid, and chlorogenic acid, are cinnamic acid derivatives. There is then alkali-catalyzed condensation of an *ortho*-hydroxyacetophenone with a benzaldehyde derivative generating chalcones and flavonones (Fig. 2), as well as a similar condensation of an *ortho*-hydroxyacetophenone with a benzoic acid derivative (acid chloride or anhydride), leading to 2-hydroxyflavanones and flavones (Heller and Forkmann 1993). The synthesis of chalcones and anthocyanidins has been described in detail by Dhar (1994). Biotransformation of flavonoids in the gut can release these cinnamic acid (phenolic acids) derivatives (Scheline 1991). In terms of their biosynthesis, the phenyl propanoid pathway produces a range of secondary metabolites such as phenolic acids, lignins, lignans and stilbenes using phenyl alanine and tyrosine as the precursor. After tannins, flavonoid glycosides are by far the most common dietary sources of flavonoids. Usually 110–121 mg/day of flavonoids has been recommended as a healthy diet for an adult (Hertog et al. 1992, 1993a, 1993b).

### Metabolism

The fate of orally and parenterally administered flavonoids in mammals was reviewed by Griffiths and Barrow (1972) and later by Hollman and Katan (1998). Considerable information is available regarding the metabolism of flavonoids in animals and to a very limited extent in humans (Hackett 1986; Scheline 1991). Hertog et al. (1992) measured the content of potentially anti-carcinogenic flavonoids of 28 vegetables, wine, and fruits frequently consumed in The Netherlands and the measured flavonoids were quercetin, kaempferol, myricetin, apigenin, and luteolin. The mean daily intake of these five anti-oxidant flavonoids was 23 mg/day, which exceeds the intake of other familiar anti-oxidants such as b-carotene (2–3 mg/day) and vitamin E (7–10 mg/day) and is about one-third the average intake of vitamin C (70–100 mg/day) (Hertog et al. 1993b). Quercetin is the most important contributor to the estimated intake of flavonoids, mainly from the consumption of apples and onions (Knekt et al. 1996; Gibellini et al. 2011; Giuliani et al. 2008). It is extremely difficult to estimate the daily human intake of flavonoids, especially because of the lack of standardized analytical methods (Scalbert and Williamson 2000). However, the average daily intake of the most abundant flavonoids, catechins, is ∼100 mg (Perez-Vizcaino et al. 2009). Similar to daily intake, it is also quite complex to assess and quantify the bioavailability of flavonoids (Russo 2007). In countries such as Japan, Korea, China, and Taiwan, the mean daily intake of soy products has been estimated to be in the range of 10–50 g compared to only 1–3 g in the United States (Messina et al. 1994).

Flavonoid pharmacokinetics is complex, since they are usually contained as glucosides in fruits and vegetables, cleaved and glucuronated during uptake. Glucuronides may be metabolized, or stored or set free as aglycones by tissue-specific glucuronidases; thus, plasma concentration may not always be a good measure of bioavailability (Seelinger et al. 2008). Most flavonoids, except catechins, exist in nature as glycosides. Moreover, at least quercetin glucosides were absorbed better than the aglycone quercetin-b-glucoside (Hollman and Katan 1998). Finally, supplementation of the diet should more appropriately use flavonoid glycosides instead of aglycones. However, this has been questioned by other researchers (Manach et al. 1997). The role of flavonoid glycosylation in facilitating absorption is questioned by the fact that catechin, which is not glycosylated in nature, is absorbed relatively efficiently (Okushio et al. 1996). Because the half-lives of conjugated flavonoids are rather long (23–28 h) (Young et al. 1999), accumulation may occur with regular intakes, which may in turn result in sufficiently active flavonoid concentrations (Nijeveldt et al. 2001). Flavanoid bioavailability and the mechanism by which flavonoids are absorbed from intestine and metabolized via microbial catabolism, conjugation in liver and enterocytes have been studied by a number of workers (Hollman and Katan 1999; Scalbert and Williamson 2000, Hollman 2004; Passamonti et al. 2009).

Studies in human and animals have indicated that some PPT (for example cinnamate conjugates, flavanols, quercetin glucosides) can be absorbed in the small intestine (Olthof et al. 2000, 2003; Nardini et al. 2002; Cermak et al. 2004) while quercetin, quercetin galactoside, rutin, naringenin-7-glucoside and many others are not. Mechanism of absorption has not been completely elucidated while the membrane transport of flavonoids is a fundamental part of their bioavailability in both plant and animal organisms, and current knowledge suggests the involvement of both ATP-dependent pumps and ATP-independent transporters (Passamonti et al. 2009).

Depending on PPT subclass, only 5–10 % of amount consumed is absorbed in small intestine and major part of that absorbed in the duodenum enters the circulation as methylated, sulfate-conjugated, glucuronide-conjugated and glycine-conjugated forms (Kroon et al. 2004). The rest 90–95 % of total PPT ingested plus any mammalian glucuronide excreted through bile pass to the colon where they are metabolized by gut microflora. Transformations may be extensive, and include the removal of sugars, removal of phenolic hydroxyls, fission of aromatic rings, hydrogenation, and metabolism to carbon dioxide, possibly via oxaloacetate (Walle et al. 2001). A substantial range of microbial metabolites has been identified, including phenols and aromatic acids, phenolic acids, or lactones possessing 0, 1, or 2 phenolic hydroxyls and up to five carbons in the side chain (Clifford and Brown 2006). The elimination half-lives are very variable, ranging from as low as 1 h (Meng et al. 2001) to values in excess of 20 h (Olthof et al. 2003).

## Flavonoid as anti-cancer agents

### Epidemiological studies

A huge number of epidemiological studies have been conducted to prove the protective effect of flavonoids against cancer. Increased consumption of lignans and greater plasma concentrations of their metabolites have been linked with reduced incidence of estrogen-related cancers in some (Pietinen et al. 2001; Dai et al. 2002; Boccardo et al. 2004; McCann et al. 2004) but not all studies, (Kilkkinen et al. 2004; Zeleniuch-Jacquotte et al. 2004) and a prospective study was equivocal (den Tonkelaar et al. 2001). It has been suggested that this inconsistency might have a genetic basis (McCann et al. 2002). Increased consumption of isoflavones has also been associated with decreased risk of estrogen-related cancers and vascular diseases (Arai et al. 2000; Birt et al. 2001). Data from four cohort studies and six case–control studies, which have examined associations of flavonoid intake with cancer risk revealed that flavonoids, especially quercetin, may reduce the risk of lung cancer in two studies but a non-significant increased risk in a third study. High versus low quercetin and kaempferol intakes were associated with 40 and 50 % reduction in risk, respectively, for stomach cancer. There was no statistically significant association of any flavonoids with either bladder cancer or breast cancer risk (Neuhouser 2004). In a network of multicentric Italian case–control studies including about 10,000 incident, histologically confirmed cases of selected cancers and over 16,000 controls, the association of flavonoids, proanthocyanidins and cancer risk was evaluated by Rossi et al. (2010). It was found that total flavonoids, flavanones, and flavonols were inversely related to oral and laryngeal cancers (ORs, respectively, 0.56 and 0.60 for total flavonoids; 0.51 and 0.60 for flavanones; and 0.62 and 0.32 for flavonols). Flavonols were also inversely related to laryngeal cancer (OR 0.64), whereas flavanones were inversely related to esophageal cancer (OR 0.38). A reduced risk of colorectal cancer was found for high intake of anthocyanidins (OR 0.67), flavonols (OR 0.64), flavones (OR 0.78), and isoflavones (OR 0.76). Inverse relations with breast cancer were found for flavones (OR 0.81) and flavonols (OR 0.80). Flavonols (OR 0.63) and isoflavones (OR 0.51) were inversely associated to ovarian cancer, whereas flavonols (OR 0.69) and flavones (OR 0.68) were inversely associated to renal cancer. No association between flavonoids and prostate cancer emerged, whereas inverse association was found between proanthocyanidins and colorectal cancer. These associations appeared stronger for proanthocyanidins with a higher degree of polymerization (Rossi et al. 2010).

In the European Prospective Investigation into Cancer and Nutrition study (Nothlings et al. 2008), the intake of vegetables, legumes, and fruit was significantly associated with reduced risks of CVD mortality and mortality due to non-CVD/non-cancer causes [RR 0.88 (95 % CI 0.81–0.95) and 0.90 (0.82–0.99), respectively] in a diabetic population comprising >10,000 individuals. High urinary excretion of both equol and enterolactone (mammalian metabolite of plant lignans) has been found to be associated with a significant decrease in breast cancer risk in an epidemiological case–control study in breast cancer patients (Ingram et al. 1997). Although this could suggest the possible importance of isoflavonoid and lignan metabolism in decreased breast cancer risk, the phytoestrogen excretion observed may just be a marker of dietary differences (Barnes 1998). Knekt and co-workers also estimated that men with higher quercetin intake had a lower lung cancer incidence, and men with higher myricetin intakes had a lower prostate cancer risk (Knekt et al. 2002).

The intake of flavonoids is not inversely related with bladder cancer or breast cancer risk in some of the studies (Garcia-Closas et al. 1999; Peterson et al. 2003). Quercetin has been reported to prevent renal cell cancer among male smokers (Wilson et al. 2009). A case–control study conducted between 1994 and 2002 in four Italian areas to study the relation between major flavonoid classes and renal cell carcinoma by Bosetti et al. 2007 revealed that flavonols and flavones were inversely related to the risk of renal cancer. A cohort of 34,651 postmenopausal cancer-free women revealed inverse relation between catechin intake and rectal cancer (Arts et al. 2002).

Population-based case–control studies carried out separately in Hawaii, Uruguay and Spain suggested an inverse association between different cancers (oral cavity, pharynx, larynx and esophagus, lungs, stomach) and total intake of flavonoids, beta-carotene and vitamin E (Le Marchand et al. 2000; Stefani et al. 1999a, b; Garcia-Closas et al. 1999). Inverse association of cholangiocarcinomas (CAC) with flavan-3-ols, anthocyanidins and total flavonoids has been reported and flavones may be inversely associated with hepatocellular carcinoma cells (HCC) risk (Lagiou et al. 2008). A statistically significant association between highest flavonoid intake and reduced risk of developing lung cancer has been reported whereby an increase in flavonoid intake of 20 mg/day was associated with a 10 % decreased risk of developing lung cancer (Tang et al. 2009).

The studies on tea, flavonoids and lung cancer risk indicated a small beneficial association, particularly among never-smokers. More well-designed cohort studies are needed to strengthen the evidence on effects of long-term exposure to physiological doses of dietary flavonoids (Arts 2008). Consumption of soy foods rich in isoflavones has been weakly associated with reduced colon and prostate cancer (Adlercreutz 2002; Guo et al. 2004; Holzbeierlein et al. 2005; Goetzl et al. 2007). A protective effect of flavonoids in association with vitamin C has been shown on esophageal cancer using data from case–control study conducted in northern Italy (Rossi et al. 2007). Flavonoid-rich diet may decrease pancreatic cancer risk in male smokers not consuming supplemental alpha-tocopherol and beta-carotene (Bobe 2008).

### In vitro studies

Isoflavonoids have biphasic effects on the proliferation of breast cancer cells in culture; at concentrations >5 mM, genistein exhibits a concentration-dependent ability to inhibit both growth factor-stimulated and estrogen-stimulated (reversed by 17b-estradiol) cell proliferation (So et al. 1997). Although genistein is a much better ligand for ERb than for the ERa (20-fold higher binding affinity) (Kuiper et al. 1997), it can also act as an estrogen agonist via both ERa and ERb in some test systems (Kuiper et al. 1998; Mueller et al. 2004). Furthermore, although genistein binds to the ligand-binding domain of ERb in a manner similar to that observed for 17β-estradiol, in the ERb–genistein complex the AF-2 helix (H12) does not adopt the normal agonist type position, but instead takes up a similar orientation to that induced by ER antagonists such as raloxifene (Pike et al. 1999). This suboptimal alignment of the transactivation helix is in keeping with the reported partial agonist activity of genistein via ERb in human kidney cell (Barkhem et al. 1998).

Anti-cancer activity of methanolic flower extract of *Tecoma stans* (METS) was evaluated by both in vitro (Vero and Hep 2 cell lines) and in vivo (using Ehrlich ascites carcinoma tumor model) methods and compared with 5-flurouracil. A significant dose-dependent anti-tumor activity was indicated (Kameshwaran et al. 2012). Enriched ginger extract exhibited higher anti-cancer activity on MCF-7 breast cancer cell lines with IC 50 value 34.8 and 25.7 μg/ml for two varieties. IC50 values for MDA-MB-231 were 32.5 and 30.2 μg/ml for rhizome extract of two varieties (Rahman et al. 2011). Luteolin-7-methyl ether isolated from leaves of *Blumea balsemifera* showed strong cytotoxicity against human lung cancer cell lines (NCI-H187) with IC 50 of 1.29 μg/ml and moderate toxicity against oral cavity cancer cell lines (KB) with IC 50 of 17.83 μg/ml (Saewan et al. 2011).

In vitro and in vivo studies on anti-cancer activity of flavonoids isolated from a herbal formulation revealed IC 50 of 24.948, 31.569 and 6.923 μg/ml, respectively, on three cancer cell lines MCF-7, Hep G-2 and ES-2 with dose-dependent inhibitory effect on hepatocellular carcinoma in mice (Liu et al. 2011). Broccolini leaf flavonoids (BLF) possess a dose-dependent anti-proliferative effects on four human cancer cell lines (SW480, HepG2, Hela, and A549) and apoptosis induction activity on SW480 cell line. Thus, the hybrid species Broccolini could be considered as a functional vegetable with potential in assisting for the treatment of four human cancers examined (Wang and Zhang 2012).

Apigenin inhibited skin papillomas and showed the tendency to decrease conversion of papillomas to carcinomas (Wei et al. 1990). Luteolin has been shown to penetrate into human skin, making it also a candidate for the prevention and treatment of skin cancer (Seelinger et al. 2008). Seufi et al. (2009) demonstrated that preventive effect of quercetin on hepato carcinomas in rats by RAPD-PCR, whereby, it was proved that quercitin exerted a preventive effect via decreased oxidative stress and decreased anti-oxidant activity. Dietary proanthocyanidins mostly present in apples, pears and pulses has been suggested to reduce the risk of pancreatic cancer by 25 % (Rossi et al. 2010).

Ethanolic extract of propolis has been found to inhibit urinary bladder transitional cell carcinoma (TCC) cell proliferation with no cytotoxic effect on normal epithelial cells (Oršolić et al. 2010). Genistein inhibited the expression of micro-RNA 21 in A-498 (RCC) cells and in the tumors formed after injecting genistein treated A-498 cells in nude mice besides inhibiting tumor formation (Zaman et al. 2012).

Kaempferol, a dietary flavonoid is effective in reducing vascular endothelial growth factor (VEGF) expression in ovarian cancer cells. It enhances the effect of cisplatin through downregulation of cMyc in promoting apoptosis of ovarian cancer cells (Luo et al. 2010). The growth of U14 cervical cancer could be inhibited by *Scutellaria baicalensis* total flavonoids (STF), the cell proliferation inhibited by arresting cell cycle and cell apoptosis induced by regulating the expression of Bax and Bcl-2 gene by treatment of STF (Peng et al. 2011). Some of the Indian medicinal plants like Ashwagandha, Curcumin, *Lithosprmum radix*, green tea, Chinese herb Astragalus and Japanese herb Juzen-Taiho-To have been reported to be effective against various cell lines of lung cancers (Ravichandiran et al. 2011). A comparison of cytotoxic effect of 11 flavonoids on chronic myeloid leukemia K562 cells and peripheral blood mononuclear cells indicated that baicalein and myricetin had a specific cytotoxic effect on leukemia cells (Romanouskaya and Grinev 2009).

Apoptotic activity of 22 flavonoids and related compounds in leukemic U937 cells were tested by Monasterio et al. (2004). They reported that flavones but none of the isoflavones induced the apoptotic cell death as determined by reduction in cell viability, flow cytometery and oligonucleosomal DNA fragmentation. The molecular consequences of apigenin treatment in myeloid and erythroid subtypes reveal the blocked proliferation of both cell lines through cell cycle arrest in different phases. JAK/STAT was one of the major target of apigenin but at the same time apigenin reduced susceptibility toward the commonly used therapeutic agent vincristine (Ruela-de-Sousa et al. 2010). A newly synthesized flavonoid III-10 could express anti-cancer effect on human U937 leukemia cell line through differentiation induction. The differentiation-related proteins phospholipids scramblase 1 (PLSCR1) and promyelocytic protein (PML) were upregulated after III-10 treatment through activation of protein kinase Cδ (Qin et al. 2012). Quercetin inhibited thyroid cell growth in association with inhibition of insulin-modulated-PI3-Kinase-AKT kinase activity. It also decreased TSH-modulated RNA level of NIS (sodium iodide sympoter) gene and thereby suggested to be a novel disrupter of thyroid function which has potential uses in thyroid cancers (Giuliani et al. 2008). Chrysin inhibited proliferation of HTH 7 and KAT 18 (anaplastic thyroid cancer cell lines) in a dose and time-dependent manner. A significant increase in cleaved caspase-3, cleaved polyADP ribose polymerase (PARP) along with a decrease in cyclin D1, Mcl-1 and XIAP was observed (Phan et al. 2011). BNF (β-napthoflavone) showed a moderate anti-proliferative activity in WHCO-6 cells and a weak activity in WHCO-1 cells. It resulted in differential expression of CYP1A1, CYP1A2 and CYP1B1 (Molepo 2010).

### In vivo studies

In vivo studies using animal models have suggested the protective effect of flavonoids against initiation as well as tumor progression. Animal model studies have provided the initial experimental evidence that soy can prevent breast cancer (Barnes 1998; Messina et al. 1994). Fermented soy milk (rich in genistein and daidzein), given to rats at 7 weeks of age, inhibited mammary tumorigenesis induced by PhIP (2-amino-1-methyl-6-phenylimidazo [4,5-b] pyridine (Ohta et al. 2000). In syngenetic mice, i.p. administration of quercetin and apigenin inhibited growth and metastatic potential of melanoma cell (B16-BL6) along with significant decrease in their invasion in vitro (Caltagirone et al. 2000). A polymethoxy flavonoid, nobiletin, inhibited the tumor-invasive activity of human fibrosarcoma HT-1080 cells in the Matrigel model through suppressing the expression of metalloproteases and augmenting of production of tissue inhibitors of metalloproteinases in tumor cells (Sato et al. 2002). Furthermore, injection of prepubertal rats with genistein (500 mg/g body weight) or estradiol benzoate (500 ng/g body weight) on days 16, 18, and 20 showed that both treatments resulted in significantly increased mammary gland terminal end buds and increased ductal branching compared to controls, indicating an ER-dependent action of genistein in mammary gland proliferation and differentiation, which could be protective against mammary cancer (Cotroneo et al. 2002). Overall, these results indicate that genistein has very complex effects on carcinogen-induced mammary cancer in the rat model and great care is required in interpreting these results and drawing parallels with human breast cancer. Equol has been found to be a novel anti-androgen that inhibits prostate growth and hormone feedback in rat studies (Lund et al. 2004). In nude mice with xenografted tumors using HAK-1B hepatoma cells, luteolin significantly inhibited the growth of the tumors in a dosage-dependent manner by targetting STAT3 through dual pathways—the ubiquitin-dependent degradation in Tyr^705^-phosphorylated STAT3 and the gradual downregulation in Ser^727^-phosphorylated STAT3 through inactivation of CDK5, thereby triggering apoptosis via upregulation in Fas/CD95 (Selvendiran et al. 2006). A flavonoid rich fraction (Fr-6) and a more purified proanthocyanidin (PAC) were isolated from cranberry and both fractions are found to slow the growth of explant tumor U87 in vivo, PAC inhibited growth of HT-29 and DU145 explants (*p* < 0.05), inducing complete regression of two DU145 tumor explants (Ferguson et al. 2004). A covalent conjugate of artemisinin (flavonoid from *Artemisia annua*) and transferrin (ART-Tf), an iron transport protein in human, are actively taken up by cancer cells through the transferrin receptor (TfR)-mediated endocytosis pathway, and show significantly higher anti-cancer activity than unconjugated artemisinin (Lai et al. 2005; Nakase et al. 2009). Like ART-Tf, artemisinin–peptide conjugates that are designed to target TfR also showed highly potent and selective anti-cancer activities (Oh et al. 2009). Although the generation of free radicals originating from the reaction of artemisinin with molecular iron is mentioned as one of the main mechanism for its anti-cancer activity, there are other mechanisms, crucial for cancer proliferation and survival that are affected by artemisinins. These mechanisms have been described in a current review (Firestone and Sundar 2009; Ferreira et al. 2010). Mammographic breast density can be used as biomarker of estrogenic or anti-estrogenic effects of a particular treatment on breast tissue (Atkinson et al. 2004). Consumption of a dietary supplement that provided red clover-derived isoflavones (26 mg biochanin A, 16 mg formononetin, 1 mg genistein, and 0.5 mg daidzein) for 12 months did not increase mammographic breast density in postmenopausal women, suggesting neither estrogenic nor anti-estrogenic effects, of this supplement at the dose given, on the breast (Atkinson et al. 2004). Honeybee propolis and its polyphenolics exerted an anti-metastatic anti-tumor effect in mice and rats and considerable cytotoxicity without cross resistance in both wild-type and chemoresistant human tumor cell lines suggesting these to be potent adjunct to chemotherapy and radiotherapy in treatment of cancers (Oršolić et al. 2010). Colon cancer risk is influenced by estrogen exposure; studies with estrogen receptor α knockout mice indicate that it may be independent of estrogen receptor α (Guo et al. 2004). Furthermore, in vivo studies in male rats have shown that genistein decreases the amount of EGF receptor present in the prostate, indicating that the observed decrease in tyrosine phosphorylation may be only a secondary effect of the influence of genistein on the expression or turnover of EGF receptor (Dalu et al. 1998). Luteolin can delay or block the development of cancer cells in vitro and in vivo by protection from carcinogenic stimuli, by inhibition of tumor cell proliferation, by induction of cell cycle arrest and by induction of apoptosis via intrinsic and extrinsic signaling pathways. When compared to other flavonoids, luteolin was usually among the most effective ones, inhibiting tumor cell proliferation with IC50 values between 3 and 50 μM in vitro and in vivo by 5–10 mg/kg i.p., intragastric application of 0.1–0.3 mg/kg/d, or as food additive in concentrations of 50–200 ppm (Seelinger et al. 2008).

## Molecular mechanism of anti-cancer effect

Flavonoids have been very often pointed out as in vitro enzyme inhibitors and ligands of receptors involved in signal transduction (Middleton and Kandaswami 1994; Havsteen 2002; Williams et al. 2004; Brown et al. 2007; Balasuriya and Rupasinghe 2011). So these flavonoid–protein interactions together with their anti-oxidant properties are the key features for their potential health benefits. Furthermore, some effects may be a result of a combination of radical scavenging and interaction with enzyme functions. The phenolic nucleus is a structural unit that is favorable to molecular (non-covalent) interactions with proteins.

These interactions can be either Vander wall or electrostatic interactions. In former type, the non-polar polarizable aromatic ring can develop strong dispersion interactions with non-polar amino acid residues followed by simultaneous release of water; while in later type, H-bonding is the most important electrostatic interaction. Flavanoid–protein redox reactions and oxidative covalent coupling may result fom one-or two-electron oxidation of the flavonoid brought about by autooxidation, scavanging of reactive oxygen species and enzymatic oxidation. For some conformationally open proteins (e.g. salivary proteins), binding constants are quite low with polyphenols but polymerization and condensation of these polyphenols produces an increase in affinity (Kurisawa et al. 2003; Kim et al. 2004). It can be due to unspecific binding along an extended protein chain or at the surface of globular proteins (Haslam 1996; Spencer et al. 1988; Xi and Guo 2008).

### Inhibition of PK_s_

Phosphorylation of proteins at OH groups of serine, threonine, and tyrosine residues is an important mechanism of intracellular signal transduction involved in various cellular responses including the regulation of cell growth and proliferation (Birt et al. 2001; Bridges 2001). The reaction makes use of ATP as a phosphate donor and is catalyzed by protein kinases. For instance, growth factor hormones bind to extracellular domains of large transmembrane receptors that display a tyrosine kinase moiety in their intracellular portion. As a consequence of hormone–receptor binding, the receptor dimerizes and becomes active in the phosphorylation of proteins close to the membrane, thereby triggering a large number of signaling pathways themselves involving other PKs, such as PKC, a Ser/Thr PK, and mitogen-activated PKs (MAPKs). On the other hand, each phase of the cell cycle, during which the DNA is replicated and the chromosomes built and then separated, is characterized by intense bursts of phosphorylation controlled by highly regulated kinases called cyclin-dependent kinases (CDKs). A possible mechanism for the potential anti-carcinogenic effects of flavonoids could be their ability to inhibit various PKs, thereby inhibiting signal transduction event of cell proliferation.

The isoflavone genistein has been shown to inhibit the epidermal growth factor (EGF) receptor in the submicromolar range by competing with ATP for its binding site (Rudrabhatla and Rajasekharan 2005; Akiyama et al. 1987). Similarly, butein (2′,3,4,4′-tetrahydroxychalcone) appears as a specific inhibitor of tyrosine kinases (IC50 for EGF receptor¼65 mM) acting competitively to ATP and non-competitively to the phosphate acceptor and having no affinity for Ser/Thr PKs such as PKC and the cAMP-dependent PKA (Yang et al. 1998, 2006).

Recently, PKC was shown to be efficiently inhibited by flavones and flavonols having a 3′,4′-dihydroxy substitution on the B ring (efficient concentrations 50 in the range 1–10 mM) (Agullo et al. 1997; Gamet-Payrastre et al. 1999) phosphoinositide 3-kinase (PI3-K), a lipid kinase catalyzing phosphorylation of inositol lipids at the D3 position of the inositol ring to form new intracellular lipid second messengers (Gamet-Payrastre et al. 1999) is also inhibited by flavonoids.

Consistently, studies with intact cells have shown that various flavonoids can cause cell cycle arrest in correlation to their ability to inhibit CDKs (Zi et al.^1998^; Casagrande and Darbon 2001). Flavonoids can also modulate the activity of MAPKs as a possible mechanism for their potential anti-neurodegenerative action (Schroeter et al. 2001, 2002) and protection against autoimmune, allergic, and cardiovascular diseases (Yoshizumi et al. 2002; Ahn et al. 2004). For instance, investigations on intact antigen-presenting dendritic cells have shown that the MAP kinases involved in cell maturation (ERK, p38 kinase, JNK) can be activated by bacterial lipopolysaccharide and that this activation is strongly inhibited by pretreatment of the cells by EGCG.61 However, no evidence is provided that the mechanism actually proceeds via direct EGCG–MAPK inhibition.

### Inhibition of prooxidant enzymes

Formation of reactive oxygen species (ROS) is a major step in the tumor promotion and progression stages. The involvement of ROS in tumor progression has been demonstrated in human cells. NADPH oxidase I (NOX 1), an enzyme that produces superoxide is overexpressed in colon and prostate cancer cell lines (Fukuyama et al. 2005; Lim et al. 2005), while its downregulation reverses tumor growth (Arnold et al. 2007). ROS play important role in DNA damaging and mutagenic signaling (Poli et al. 2004; Valko et al. 2004). ROS act as secondary messenger in several pathways that lead to increase in cell proliferation, resistance to apoptosis, activation of proto-oncogenes such as cFOS, cJUN and cMyc. In human hepatoma cells, ROS modulate the expression of cFOS and cJUN through PKB pathway (Liu et al. 2002).

Lipoxygenases (LOX), cycloxygenases (COXs), and xanthine oxidase (XO) are metalloenzymes whose catalytic cycle involves ROS such as lipid peroxyl radicals, superoxide, and hydrogen peroxide. LOXs and COXs catalyze important steps in the biosynthesis of leucotrienes and prostaglandins from arachidonic acid, which is an important cascade in the development of inflammatory responses. XO catalyzes the ultimate step in purine biosynthesis, i.e., the conversion of xanthine into uric acid. XO inhibition is an important issue in the treatment of gout. Flavonoids may exert part of their anti-oxidant and anti-inflammatory activities via direct inhibition of these prooxidant enzymes (LOXs, COXs, and XO). Similarly, flavonoids can inhibit ornithine decarboxylase (rate-limiting enzyme in polyamine biosynthesis) induced by tumor promoters, and thereby inhibiting proliferation. Typically, interpretation of the inhibition studies is complicated because of the possible combination of distinct inhibition mechanisms: formation of non-covalent enzyme-inhibitor complexes, direct scavenging by flavonoid anti-oxidants of ROS inside or outside the catalytic pocket (with simultaneous oxidation of the flavonoids), chelation of the enzyme metal centers by the flavonoids, and enzyme inactivation by reactive aryloxyl radicals, quinones, or quinonoid compounds produced upon flavonoid oxidation that may eventually form covalent adducts with the enzyme (Olivier and Claire 2005; Sandhar et al. 2011).

### Modulate the metabolism of carcinogen

Activation of a procarcinogen to carcinogen is an important step in carcinogenesis and can be modulated by flavonoids. Flavonoids can exert their effect by two possible mechanisms. Firstly, by interacting with phase 1 enzymes (CyP_450_) that are involved in metabolic activation of procarcinogens. Second mode of action can be the detoxification and elimination of carcinogens via induction of phase II enzymes such as UDP-glucuronyl transferase, quinone reductase and glutathione *S*-transferase.

These heme-containing cytochrome P450 (CYP) monooxygenases include several isoforms (CYP 1A1, 1A2, 1B1, 3A4, 3A5, etc.) with different tissue distributions and play a key role in the metabolism of endogenous substrates (e.g., steroids) and xenobiotics (food components, drugs, carcinogens, pollutants) (Anzenbacher and Anzenbacherová 2011; Hodek et al. 2002; Ahmad and Mukhtar 2004). Indeed, CYPs are responsible for the conversion of some procarcinogens (e.g., polyaromatic hydrocarbons or PAHs) into carcinogens (e.g., PAH epoxides). Cytochrome P450 enzymes are a good example of proteins whose function can be regulated by flavonoids via such diverse mechanisms (Hodek et al. 2002, 2009).

CYP–flavonoid interactions are a good example of the multiple ways flavonoids can affect enzymatic activities, i.e., from the regulation of gene expression to direct binding to the processed enzymes. Flavonoids can induce, or eventually inhibit, the biosynthesis of CYP 1A1 via interactions with the aryl hydrocarbon receptor (AhR), a cytosolic protein that, once activated by a ligand, translocates to the nucleus and, in association with the AhR translocator, forms a transcription factor for CYP 1A1. For instance, in human breast cancer cells, quercetin binds to AhR as an agonist and stimulates gene expression for CYP 1A1 with a parallel increase in CYP 1A1-mediated *O*-deethylation of 7-ethoxyresorufin (Ciolino et al. 1999). This process is strikingly dependent on the hydroxylation pattern of the B ring since kaempferol (3′-deoxyquercetin) binds AhR as an antagonist (no subsequent activation of CYP 1A1). It is also highly dependent on the cell type since, in hepatic cells, quercetin binds to AhR as an antagonist, thereby inhibiting gene expression for CYP 1A1 and benzo[a]pyrene activation (Kang et al. 1999). This provides a possible mechanism for the anti-cancer activity of quercetin. Flavonoids, especially flavones and flavonols, also directly bind to several CYP isoforms (1A1, 1A2, 1B1, 3A4) involved in xenobiotics metabolism and inhibit enzyme activity. Structure–activity relationships (Doostdar et al. 2000) show rather high isoform selectivities depending on the flavonoid substitution pattern and contrasted inhibition mechanisms. Finally, flavonoids are also able to inhibit CYP19 or aromatase, an enzyme catalyzing a three-step oxidation sequence resulting in aromatization of the A ring of male steroid hormones (androgens) to yield estrogens. Together with flavonoid–estrogen receptor binding, this process could be relevant to the prevention of hormone-dependent breast cancer by flavonoids (Brueggemeier et al. 2001).

Interestingly, 17β-hydroxysteroid dehydrogenase, another redox enzyme involved in steroid metabolism, is also strongly inhibited by 7-hydroxyflavonoids (Le Bail et al. 1998). For instance, the flavone apigenin is more potent at inhibiting 17b-hydroxysteroid dehydrogenase (IC50 ¼0.3 mM) than aromatase (IC50 ¼2.9 mM) and the isoflavone genistein, which is only a weak aromatase inhibitor, inhibits 17b-hydroxysteroid dehydrogenase with an IC50 of 1 mM. The flavonols quercetin, kaempferol, myricetin, the flavone apigenin, and the biflavone biapigenin were also reported to inhibit CYP3A4 activity at low micromolar concentrations in human liver microsomes (von Moltke et al. 2004).

Xenobiotics, drugs and flavonoids follow the same course of metabolism. As these compounds are hydrophobic in nature, so the first step involving the conjugation of these drugs to increase their hydrophilicity is a key step in their metabolism. This step is performed by the above mentioned phase-II enzymes. Flavonoids have been demonstrated to activate these enzymes and thereby increase detoxification and elimination of carcinogens from the body. UDPglucuronosyltransferases (UGT) use UDP-glucuronic acid as a cosubstrate and transfer glucuronic acid to available substrates thereby making them more water soluble and facilitating their excretion in the urine or bile. Similarly, sulfotransferases (SULT) catalyze the transfer of a sulfonate group, glutathione *S*-transferases (GST) transfer glutathione and *N*-acetyltransferases transfer acetyl moiety to an appropriate substrate. It has been shown that all these phase II enzymes are affected by flavonoids in cell and animal models.

An increase in mRNA levels, protein expression and enzyme activity of UGT isoform 1A1 has been reported in human Hep G2 cells and human colon carcinoma cells by chrysin, apigenin, baicalein, diosmetin, quercetin and kaempferol (Galijatovic et al. 2001; Walle and Walle 2002; Sugatani et al. 2004). van der Logt et al. 2003 reported an increase in UGT activity in intestine and liver by feeding rats on a diet containing 1 % wt/wt quercetin and 0.5 % wt/wt flavones. Quercetin significantly increased UGT1A1 mRNA in shed enterocytes on in vivo perfusion of human jejunam and in Caco-2 cells (Petri et al. 2003). A number of flavonoids including fisetin, galangin, quercetin, kaempferol, and genistein represent potent non-competitive inhibitors of sulfotransferase 1A1 (or P-PST); this may represent an important mechanism for the chemoprevention of sulfation-induced carcinogenesis (Moon et al. 2006). Dietary intake of citrus limonoids (Perez et al. 2010) may provide a protective effect against the onset of various cancers by inducing the activity of certain phase II detoxifying enzymes [glutathione *S*-transferase and NAD(P)H: quinone reductase] in specific organs. Johnson et al. (2009) analyzed the ability of eriodictyol to activate Nrf2 and induce the phase II proteins, heme-oxygenase (HO-1), NAD(P)H: quinone oxidoreductase 1 (NQO-1), and the cellular anti-oxidant glutathione, GSH. They have shown that ARPE-19 cells that overexpress HO-1 or NQO-1 were more resistant to oxidative stress-induced cell death than control cells. Cermak (2008) reviewed flavonoids as potent inhibitors of cytosolic SULT isoforms. Quercetin inhibited sulfation of 4-nitrophenol and of several drugs including salbutamol, minoxidil, paracetamol, apomorphine in human liver cytosol (De Santi et al. 2002; Marchetti et al. 2001; Vietri et al. 2002). Quercetin significantly affected the activity of hepatic GST, whereas dietary catechin significantly changed NAD(P)H quinone oxidoreductase-1 activity (26 %) in rats. Changes in GST and NAD(P)H quinone oxidoreductase-1 activity were partly reflected on mRNA levels (Wiegand et al. 2009).

### Inhibition of multidrug resistance

Cancer cells typically overexpress P-glucoprotein (Pgp) or multidrug resistance associated protein (MRP) which is ATP-dependent transmembrane transporters capable of expelling a wide variety of chemically unrelated drugs used in cancer therapy at the expense of ATP hydrolysis. This phenomenon is known as multidrug resistance (MDR) and inhibition of MDR, to prevent drug efflux has potential clinical application during cancer therapy.

Quercetin was shown to efficiently inhibit the Pgp-mediated drug efflux by inhibiting the overexpression of human MDR1 gene (Kioka et al. 1992) and by inhibiting the ATPase activity required for transport (Shapiro and Ling 1997). From investigations using a soluble cytosolic portion of mouse Pgp, which includes the nucleotide- and drug-binding domains, it was possible to monitor flavonoid binding by fluorescence as well as its influence on ATP binding and the efflux of the anti-cancer steroid drug RU 486 (Conseil et al. 1998). Flavones (aglycones) bearing OH groups at positions 3 and 5 come up as efficient mouse Pgp ligands with apparent dissociation constants lower than 10 mM. By contrast, the quercetin glycoside rutin, the flavanone naringenin, and the isoflavone genistein have low affinity for Pgp. Interestingly, flavones and flavonols behave as bifunctional inhibitors whose binding site overlap the vicinal binding sites for both ATP and RU 486. Those trends were confirmed using a cytosolic portion of Pgp from the parasite *Leishmania tropica* (Perez-Victoria et al. 1999). Flavonoids have been reported to inhibit ATPase activity, nucleotide hydrolysis and energy-dependent drug interaction with transporter enriched membranes (Di Pietro et al. 2002). This unique property of reversal of MDR has been found to enhance doxorubin (DOX)-induced anti-tumor activity by increasing the DOX concentration at target site (Blagosklonny 2001). To study that how epicatechin gallate, epigallocatechin gallate, genistein, genistin, naringenin, naringin, quercetin and xanthohumol will modulate cellular uptake and permeability [P(e)] of multidrug-resistant substrates, cyclosporin A (CSA) and digoxin, across Caco-2 and MDCKII-MDR1 cell-transport models, uptake experiments were perfomed with and without flavonids. Aglycone flavonoids reduced the P(e) of CSA to a greater extent than that of digoxin, suggesting that transport mechanism of CSA can be different from digoxin (Rodriguez-Proteau et al. 2006). Ofer et al. (2006) compared the potency of quercetin, isoquercitin, spiraeoside, rutin, kaempferol, naringenin, naringin and hesperetin to inhibit the transport through P-gp transporters (substrate ^3^H-talinolol) and OCT (substrate ^14^C-TEA) of Caco-2 cells and LLC-PK 1 cells, respectively. Six of the investigated flavonoids reduced the secretory flux of talinolol across Caco-2 cells but none of the selected flavonoids was able to replace ^3^H-talinolol from its binding to P-gp. This might be due to an interaction with P-gp, but apparently not via competition at the talinolol binding site of P-gp. The investigated flavonoids did show potency to inhibit OCT-mediated transport (IC50-values: quercetin < kaempferol ≪ naringenin < isoquercitrin < spiraeoside ≪ rutin < hesperetin < naringin) may be by the inhibition of members of the OCT family.

This flavonoid–ABC-transporter interaction could be beneficial for poorly absorbed drugs but could also result in severe drug intoxication, especially drugs with a narrow therapeutic window (Alvarez et al. 2010). Most of the studies have demonstrated inhibitory effects of flavonoids on the substrate efflux in cells that either endogenously expressed these transporters or that were transfected with them (Morris and Zhang 2006). These ABC-transporter proteins can affect the oral availability and tissue distribution of the flavonoids also, thereby modifying their beneficial effects (Brand et al. 2006).

### Anti-oxidant properties

In addition to enzymatic oxidation, flavonoid oxidation can take place via autoxidation (metal-catalyzed oxidation by dioxygen) and ROS scavenging. The former process can be related to flavonoid cytotoxicity (ROS production) while the latter is one of the main anti-oxidant mechanisms. Both processes may be modulated by flavonoid–protein binding. Although poorly documented so far, these points could be important and, for instance, albumin–flavonoid complexes with an affinity for LDL could act as the true plasma anti-oxidants participating in the regeneration of α-tocopherol from the α-tocopheryl radical formed upon scavenging of LDL-bound lipid peroxyl radicals. In addition, flavonoid–protein complexation can be expected to provide protection to the protein against oxidative degradation.

Since lipid peroxidation is clearly related to the onset of atherosclerosis and the impairment of membrane functions, the influence of proteins on the ability of flavonoids to inhibit lipid peroxidation deserves examination (Peng and Kuo 2003; Liu et al. 2006; Kumar et al. 2008; Li 2011). Such investigations have been carried out with BSA and lecithin liposomes (Heinonen et al. 1998; Sivonová et al. 2006). Whereas BSA alone already slows down the formation of lipid hydroperoxides and hexanal, its influence on the anti-peroxidizing activity of the selected polyphenols is highly dependent on the polyphenolic structure. Hence, BSA lowers the inhibition of hydroperoxide formation by catechins and caffeic acid, enhances inhibition by malvidin and rutin, and leaves essentially unchanged inhibition by quercetin. No clear interpretation based on polyphenol–BSA binding can be given. One-electron oxidation of protein-bound phenols to form reactive aryloxyl radicals is a possible pro-oxidant mechanism, since these radicals can propagate H-atom or electron transfers within the protein. In addition to phenol–protein covalent coupling, these phenol-mediated oxidative damages to proteins could be detrimental to their function as enzymes, receptors, and membrane transporters.

Anti-oxidant properties have been reported for isoflavones both in vitro and in vivo (Wei et al. 1995; Wiseman et al. 1998, 2000; Mitchell et al. 1998). Equol, in model membrane systems, was a more effective anti-oxidant than genistein or the parent compound daidzein (Wiseman et al. 1998) and shows structural similarity to the tocopherols. Daidzein and geinstein showed anti-oxidant action in primary and cancer lymphocytes (Jurkat cells), both isoflavones increased DNA protection against oxidative damage and decreased lipid peroxidation (Foti et al. 2005). Moreover, a protective effect was achieved at concentrations that can be achieved in plasma following soy consumption. An important aspect of cancer risk is the involvement of the inflammatory response, which involves the production of cytokines and proinflammatory oxidants such as the hypochlorous acid produced by neutrophils and peroxynitrite by macrophages, which react with phenolic tyrosine residues on proteins to form chloro- and nitrotyrosine (D’Alessandro et al. 2003). It has been reported that neutrophil myleloperoxidase chlorinates and nitrates isoflavones and enhances their anti-oxidant properties, thus soy isoflavones may have potentially protective benefits at sites of inflammation (D’Alessandro et al. 2003). Anti-oxidant action could also contribute to anti-cancer ability because ROS could initiate signal transduction through the mitogen-activated protein (MAP) kinases (Wiseman and Halliwell 1996). There have been a number of reports relating to the possible anti-oxidant effects of isoflavone consumption. Soy isoflavone consumption as a soy protein burger (56 mg isoflavones/day for 17 days) decreased plasma F2-isoprostane concentrations in healthy young adults (Wiseman et al. 2000). Consumption of a soy isoflavone supplement (50 mg isoflavones, twice a day for 3 weeks) decreased a biomarker of DNA oxidative damage (white cell 5-hydroxymethyl-2′-deoxyuridine concentrations) but did not alter plasma F2-isoprostane concentrations (Djuric et al. 2001). Furthermore, consumption of soy protein (110 mg isoflavones/day for 4 weeks) decreased plasma peroxide concentrations and increased total anti-oxidant status but did not effect a biomarker of oxidative DNA damage (Bazzoli et al. 2002). It is of considerable interest that widely differing effects in relation to the potential benefits to human health are frequently reported for isoflavones consumed within the food matrix in soy foods, compared to those consumed in capsule or tablet form as dietary supplements.

### Anti-angiogenesis

Angiogenesis, the formation of new blood vessels, is an important process which is regulated by endogenous angiogenic and angiostatic factors. Any alteration in this tightly regulated process can lead to a persistent and uncontrolled growth and metastasis of tumors. Flavanoids have been reported as angiogenesis inhibitors (Tosetti et al. 2002). These inhibitors can cause lack of diffusion of nutrients and oxygen to rapidly growing cancerous cells due to anti-angiogenic properties and hence lead to cell death. Angiogenesis inhibitors can interfere with various steps in angiogenesis, such as the proliferation and migration of endothelial cells and lumen formation. A possible mechanism could be inhibition of protein kinases (Oikawa et al. 1992). These enzymes are implicated to play an important role in signal transduction and are known for their effects on angiogenesis. Genistein is a potent inhibitor of angiogenesis in vitro (Fotsis et al. 1993; Kim 2003; Su et al. 2005) and thus could have therapeutic applications in the treatment of chronic neovascular diseases including solid tumor growth (Fotsis et al. 1995) and inhibition of neovascularization of the eye by genistein has been reported (Kruse et al. 1997; Joussen et al. 2000; Xu and Yuan 2009). Studies on the inhibition of cell proliferation and angiogenesis by flavonoids in six different cancer cell lines had been reported and noted that the IC50 of active flavonoids were in the low micromolar range, physiologically available concentrations (Fotsis et al. 1997). Isoflavonones (genistein, genistin, daidzein, and biochanin A) also inhibit growth of murine and human bladder cancer cell lines by inducing cell cycle arrest, apoptosis, and angiogenesis (Zhou et al. 1998). Luteolin has been found to inhibit VEGF-induced angiogenesis; inhibition of endothelial cell survival and proliferation by targeting phosphatidylinositol-3-kinase action (Bagli et al. 2004). Favot et al. (2003) suggested the involvement of cyclin-dependent pathway in the inhibitory effect of delphinidin on angiogenesis. During the last decade, some more novel molecular targets for the inhibition of angiogenesis by genistein have been discovered including tissue factor, endostatin, and angiostatin (Su et al. 2005).

Genistein may enhance the action of transforming growth factor-β (TGF-β) (Kim et al. 1998; Sathyamoorthy et al. 2005). This action may be a link between the effects of genistein in a variety of chronic diseases (Barnes 1998) including atherosclerosis and hereditary hemorrhagic telangiectasia (the Osler–Weber–Rendu syndrome) in which defects in TGF-β have been characterized (Johnson et al. 1996). Schindler and Mentlein (2006) determined whether secondary plant constituents, i.e., flavonoids, tocopherols, curcumin, and other substances regulate VEGF in human tumor cells in vitro. It was found that the glycosylated flavonoids (i.e., naringin, a constituent of citrus fruits, and rutin, a constituent of cranberries) induced the greatest response to treatment at the lowest concentration in MDA human breast cancer cells. Inhibition of VEGF release by flavonoids, tocopherols, and lovastatin in models of neoplastic cells suggests a novel mechanism for mammary cancer prevention. He et al. (2011) indicate that hispidulin targets the VEGF receptor 2-mediated PI3K/Akt/mTOR signaling pathway in endothelial cells, leading to the suppression of pancreatic tumor growth and angiogenesis.

### Induce apoptosis and cell cycle arrest

Apoptosis is a programed cell death to eliminate damaged or unwanted cells. It is regulated by a variety of genes that can either promote apoptosis or can favor cell survival in response to internal or external stimuli. Dysregulation of apoptosis could play a critical role in oncogenesis. Among these genes, the tumor suppressor p53 plays a pivotal role in controlling the cell cycle, apoptosis, genomic integrity, and DNArepair (Bode and Dong 2004) by acting as transactivator or as transrepressor (Ho et al. 2005). After activation, p53 can bind to regulatory DNA sequences and activate the expression of genes involved in cell cycle inhibition (p21, reprimo, cyclin G1, GADD45), apoptosis (PERP, NOXA, PUMA, p53AIP1, ASPP1/2, Fas, BAX, PIDD) and genetic stability (p21, DDB2, MSH2, XPC) (Carr 2000; Amin et al. 2009). EGCG also activated p53 and BAX in breast carcinoma cells (Roy et al. 2005). Genistein induced G2/M arrest and apoptosis in human malignant glioma cell lines by activating p53 and p21 (Schmidt et al. 2008). In addition to p53, mammalian cells contain two closely related proteins, p63 and p73. EGCG induces apoptosis by activating p73-dependent expression of a subset of p53 target genes (Amin et al. 2007).

Nuclear factor-kappa B (NF-кB) family of transcription factors consists of five members, p50, p52, p65 (Rel A), c-Rel, and Rel B, which share an N-terminal Rel homology domain responsible for DNA binding. NF-кB is activated by free radicals, inflammatory stimuli, cytokines, carcinogens, tumor promoters, endotoxins, γ-radiation, ultraviolet (UV) light, and X-rays and induces NF-кB target genes important for cellular growth and transformation, suppression of apoptosis, invasion, metastasis, chemoresistance, radioresistance, and inflammation. Flavonoids may block one or more steps in the NF-кB signaling pathway such as inhibition of the most upstream growth factor receptors that activate the NF-кB signaling cascade, translocation of NF-кB to the nucleus, DNA binding of the dimers, or interactions with the basal transcriptional machinery (Ju et al. 2007). The NF-кB target genes influenced by the flavonoids include inhibition of Bcl-2 and Bclx(L), cyclin D1, matrix metalloproteinases (MMP), and VEGF (Hastak et al. 2003; Li et al. 2006).

Flavonoids have been found to suppress activator protein-1 (AP-1) activation and modulate AP-1 target genes. AP-1 is a group of dimeric leucine zipper proteins consisting of Jun (c-Jun, JunB, JunD), Fos (c-Fos, FosB, Fra-1, and Fra-2), Maf (c-Maf, MafB, MafA, MafG/F/K, and Nrl), and ATF (ATF2, LRF1/ATF3, B-ATF, JDP1, JDP2) subfamilies (Shaulian and Karin 2002). These proteins form either homo- or heterodimers and bind either to AP-1 DNA recognition elements (5′-TGAG/CTCA-3′) or to cAMP response elements (5′-TGACGTCA-3′) and activate their target genes. Some of the biologic effects of AP-1 are mediated by gene repression. AP-1-regulated genes include important modulators of invasion and metastasis, angiogenesis, proliferation, differentiation, and survival.

Activation of various tyrosine kinases leads to phosphorylation, dimerization, and nuclear localization of the signal transducers and activators of transcription (STAT) proteins, binding to specific DNA elements and direct transcription. Constitutive activation of STAT3 and STAT5 has been implicated in multiple myelomas, lymphomas, leukemias, and several solid tumors. Selvendiran et al. (2006) reported that luteolin inhibited phosphorylation of STAT3, which targeted it for proteosomal degradation and inhibited the expression of cyclin D1, survivin, Bcl-x(L), and VEGF.

Luteolin is capable of inducing anti-cancer effects by inducing cell cycle arrest or apoptosis in oral squamous cancer cells (Yang et al. 2008), human esophageal, lung, liver and colon cancer cells (Zhang et al. 2008; Ju et al. 2007). Luteolin inhibited proliferation and induced apoptosis of prostate cancer cells in vitro and in xenografts (Chiu and Lin 2008), with increased efficacy of cisplatin in gastric cancer cells (Wu et al. 2008). Genistein inhibited P Ca cell growth in culture by inducing G2/M arrest and apoptosis, increased the radiation efficacy against prostate cancer in cell culture and in vivo models (Lakshman et al. 2008).

Quercetin has been reported to suppress the viability of HeLa cells in a dose-dependent manner by inducing G2/M phase cell cycle arrest and mitochondrial apoptosis through a p53-dependent mechanism (Vidya et al. 2010). Some characteristic changes in nuclear morphology, phosphatidylserine externalization, mitochondrial membrane depolarization, modulation of cell-cycle regulatory proteins and NF-κB family members, upregulation of proapoptotic Bcl-2 family proteins, cytochrome C, Apaf-1 and caspases, and downregulation of anti-apoptotic Bcl-2 proteins and surviving was reported. Similarly fisetin inhibited the proliferation of bladder cancer cells by inducing apoptosis and blocking cell-cycle progression in the G0/G1 phase. It significantly increases the expression of p53 and p21 proteins, and decreases the levels of cyclin D1, cyclin A, CDK4 and CDK2, thereby contributing to cell cycle arrest. In addition, fisetin increased the expression of Bax and Bak but decreased the levels of Bcl-2 and Bcl-xL and subsequently triggered mitochondrial apoptotic pathway (Li et al. 2011a, b). Pretreatment with chrysin could increase TRAIL-induced degradation of caspase 3, caspase 8, PARP protein. Z-VAD- fmk, which is a pan-cascade inhibitor, could inhibit the apoptosis enhanced by combination of chrysin and TRAIL (Li et al. 2011a, b). 2,2 dihydroxychalcone (DHC) and fisetin, induced S and G2 phase cell-cycle arrest in LNCaP and PC3 prostate cancer cells by downregulation in gene expression of 75 key cell cycle (G2 and M phases) and enhanced expression of 50 stress response gene. Thereby, DHC and fisetin induced apoptosis, but not accelerated senescence in prostate cells (Haddad 2008). The study on diethyl 5,7,4′-trihydroxy flavanone *N*-phenyl hydrazone (N101-2), a synthesized naringenin derivative exhibited cervical cancer cell growth inhibition by arresting the cell cycle at sub-G1 phase, activating mitochondria-emanated intrinsic and Fas-mediated extrinsic signaling pathways, and inhibiting the PI3K/AKT pathway in CaSki and SiHa human cervical cancer cells (Kim et al. 2012).

## Summary and conclusions

Flavonoids greatly influence the cascade of immunological events associated with the development and progression of cancer. One has to understand the mechanism how these flavonoids get accumulated in cellular organelles and tissues once they enter inside. Flavonoids have the potential of modulatng many biological events in cancer such as apoptosis, vascularization, cell differentiation, cell proliferation, etc. A strong correlation persists between flavonoid-induced modulation of kinases with apoptosis, cell proliferation and tumor cell invasive behavior in vitro. Also, some of the dietary flavonoids have been known to display in vivo anti-tumor activity and repress in vivo angiogenesis. The cross talk between flavonoids and the key enzymes related to neoplastic cells and metastasis has to be understood in vitro and in vivo as well, providing new insights for fighting against cancer.
